# Reexamination of the UN10 Rule to Discontinue Resuscitation During In-Hospital Cardiac Arrest

**DOI:** 10.1001/jamanetworkopen.2019.4941

**Published:** 2019-05-31

**Authors:** Bradley J. Petek, Daniel N. Bennett, Christian Ngo, Paul S. Chan, Brahmajee K. Nallamothu, Steven M. Bradley, Yuanyuan Tang, Rodney A. Hayward, Carl van Walraven, Zachary D. Goldberger

**Affiliations:** 1 Massachusetts General Hospital, Boston; 2 Southwestern School of Medicine, University of Texas, Dallas; 3 University of Washington School of Medicine, Seattle; 4 Saint Luke’s Mid America Heart Institute, Kansas City, Missouri; 5 Veterans Affairs Health Services Research and Development Center of Innovation, Veterans Affairs Ann Arbor Healthcare System, Ann Arbor, Michigan; 6 Michigan Integrated Center for Health Analytics and Medical Prediction, Department of Internal Medicine, University of Michigan, Ann Arbor; 7 Center for Healthcare Delivery Innovation, Minneapolis Heart Institute, Minneapolis, Minnesota; 8 University of Ottawa, Ottawa, Ontario, Canada; 9 University of Wisconsin School of Medicine and Public Health, Madison

## Abstract

**Importance:**

Several clinical decision rules (CDRs) have been developed to help practitioners know when to safely terminate resuscitative efforts after in-hospital cardiac arrest (IHCA). The UN10 rule, a CDR that uses 3 intra-arrest variables, has been shown to predict a poor chance of survival to discharge. However, its large-scale applicability in clinical settings remains unknown.

**Objective:**

To assess the performance of a parsimonious CDR in a national cohort of individuals with IHCA.

**Design, Setting, and Participants:**

This retrospective cohort study used a nationwide cohort from the American Heart Association Get With the Guidelines–Resuscitation IHCA registry to derive a sample of 96 509 patients from 716 US hospitals who experienced IHCA from January 1, 2000, to January 26, 2016. Data analysis began in January 2018 and concluded in June 2018.

**Exposures:**

The UN10 rule uses 3 variables: (1) unwitnessed arrest, (2) nonshockable rhythm, and (3) no return of spontaneous circulation within 10 minutes of resuscitative efforts. The CDR indicates futility if all 3 criteria are met. This CDR was analyzed according to the Consolidated Health Economic Evaluation Reporting Standards (CHEERS) reporting guideline.

**Main Outcomes and Measures:**

The primary outcome was survival to hospital discharge following resuscitation. Favorable neurologic status at discharge was also assessed. Overall rates of survival and survival with favorable neurologic status (cerebral performance category score, 1 or 2) were compared with predicted values by the UN10 rule using 2 × 2 contingency tables.

**Results:**

Of 96 509 patients, 55 761 (57.8%) were men, and the mean (SD) age was 67.1 (15.3) years. In total, 18 713 patients (19.4%) survived to discharge, and 16 134 patients (16.7%) were discharged with a favorable neurologic status. Overall, 15 838 patients (16.4%) met all 3 criteria for futility in the UN10 rule. A total of 1005 patients (6.3%) who met the UN10 rule survived to discharge, and 754 (4.8%) survived with favorable neurologic status. The percentage of patients meeting the UN10 rule (ie, predicting futile resuscitation) who actually survived in our study cohort was substantially higher than the initial derivation cohort (0%) and single-center validation cohort (1.1%). The positive predictive value of the UN10 rule was 93.7% (95% CI, 93.3%-94.0%), which was lower than the initial derivation cohort (100%; 95% CI, 97.5%-100%) and validation cohort (98.9%; 95% CI, 96.5%-99.7%).

**Conclusions and Relevance:**

Patients who met the UN10 rule were associated with unfavorable neurologic status and low rates of survival after IHCA. Yet their survival rates are higher than reported in the initial validation study, raising the question of whether the UN10 rule may have limited utility as a definitive measure of futility during resuscitations in real-world clinical settings.

## Key Points

**Question:**

How does a previously developed clinical decision rule (termed the *UN10 rule*), designed to predict futility during in-hospital cardiac arrest, perform in a large national sample?

**Findings:**

In this cohort study of 96 509 patients with in-hospital cardiac arrest, the percentage of patients who satisfied the UN10 rule for futility and survived (6.3%) was substantially higher than the initial derivation cohort (0%) and single-center validation cohort (1.1%).

**Meaning:**

A revalidation study of the UN10 rule in a large cohort demonstrated moderate predictive ability to identify patients with poor survival; however, survival rates of patients who met all UN10 criteria were much higher than previous studies.

## Teaser

This cohort study assesses the performance of the UN10 clinical decision rule to predict futility during in-hospital cardiac arrest in a US national sample of patients who experienced in-hospital cardiac arrest.

## Introduction

Several clinical decision rules (CDRs) have been developed to help practitioners avoid potentially futile resuscitative efforts in hospitalized patients.^1,2,3,4,5,6,7,8^ However, their overall utility is limited, primarily because of model complexity, inadequate validation, or insufficiently low positive predictive values.^1,2,3,4,5,6^ Van Walraven et al^7,8^ developed a parsimonious model incorporating 3 readily available intra-arrest variables, to identify patients with in-hospital cardiac arrest (IHCA) who have no chance of survival to discharge. This model, which we call the *UN10 rule* based on the 3 variables (U, unwitnessed arrest; N, nonshockable rhythm; and 10, return of spontaneous circulation [ROSC] not obtained within 10 minutes), was prospectively validated in 2181 patients at a single hospital nearly 20 years ago. While it is unclear how widely used this model currently is in clinical settings, the application of a simple CDR relying on just 3 intra-arrest variables in code settings could greatly enhance termination decisions. How it performs in a broader sample of hospitalized patients and in the context of a diverse population and contemporary resuscitation care practices remains unknown.

## Methods

### Data Source

We used the American Heart Association Get With the Guidelines–Resuscitation (GWTG-R) registry, a large, multicenter, prospective, observational registry of IHCA in the United States. The registry has been described in detail previously.^9^ Briefly, trained personnel at participating hospitals record observational data during resuscitation of IHCAs, defined as apnea, absence of central palpable pulse, and unresponsiveness. Cases are identified by available arrest flow sheets, paging system logs, medication administration records, emergency resuscitation equipment, and hospital billing sheets. Information is standardized using Utstein definitions as developed by international experts.^9^ The American Heart Association provides oversight for the entire process of data collection, analysis, and reporting. A deidentified database was used for statistical analyses. The institutional review board of the University of Michigan reviewed the study protocol and determined the study was exempt. Patient consent was waived owing to the use of a deidentified database. This study follows the Consolidated Health Economic Evaluation Reporting Standards (CHEERS) reporting guideline.^10^

### Definitions

Duration of resuscitation was documented in integer minutes and was defined as the time from the onset of resuscitation to ROSC or termination of efforts when the patient was declared deceased. Return of spontaneous circulation was defined as the restoration of a pulse for at least 20 minutes during the cardiac arrest. Shockable rhythms were defined as arrests due to pulseless ventricular tachycardia or ventricular fibrillation. Nonshockable rhythms were defined as pulseless electrical activity or asystole. We used cerebral performance category (CPC) scores to assess neurologic status of survivors at the time of discharge (1, little to no major neurologic disability; 2, moderate disability; 3, severe disability; 4, coma or vegetative state; and 5, brain death).^11^ In keeping with prior literature, favorable neurologic survival was defined as survival without severe neurologic disability (ie, CPC score, 1 or 2).^12^

### Main Outcomes

The primary outcome of this validation study was survival to discharge. A patient was predicted to have no chance of survival to discharge if all 3 of the following conditions were met: (1) unwitnessed arrest (ie, not in person or by monitor), (2) a nonshockable initial rhythm (ie, pulseless electrical activity or asystole), and (3) no ROSC within 10 minutes of starting chest compressions. As previously, these 3 variables, initially derived and validated by van Walraven et al^7,8^ will be defined as the UN10 rule. Additionally, we assessed whether these 3 variables predicted survival with a favorable neurologic status. Because some percentage of survivors had missing information on CPC scores at discharge and were assumed to be missing at random, we performed multiple imputation and pooled the results with 20 data sets. Results with and without imputation were not meaningfully different.

### Study Population

We identified 197 650 patients 18 years or older with complete clinical and demographic data who experienced an index cardiac arrest at 1 of 725 study hospitals between January 1, 2000, and January 26, 2016 (Figure). After several exclusions, including 4340 individuals with cardiac arrests whose duration prior to achieving ROSC was less than 2 minutes (to ensure a veritable resuscitative effort) as well as 5355 patients who did not achieve ROSC and received less than 10 minutes of attempted resuscitation (to whom the UN10 rule would not apply), our final study population consisted of 96 509 patients with an index IHCA from 716 hospitals. A total of 2827 survivors (15.1%) had missing information on CPC scores at discharge.

**Figure.**
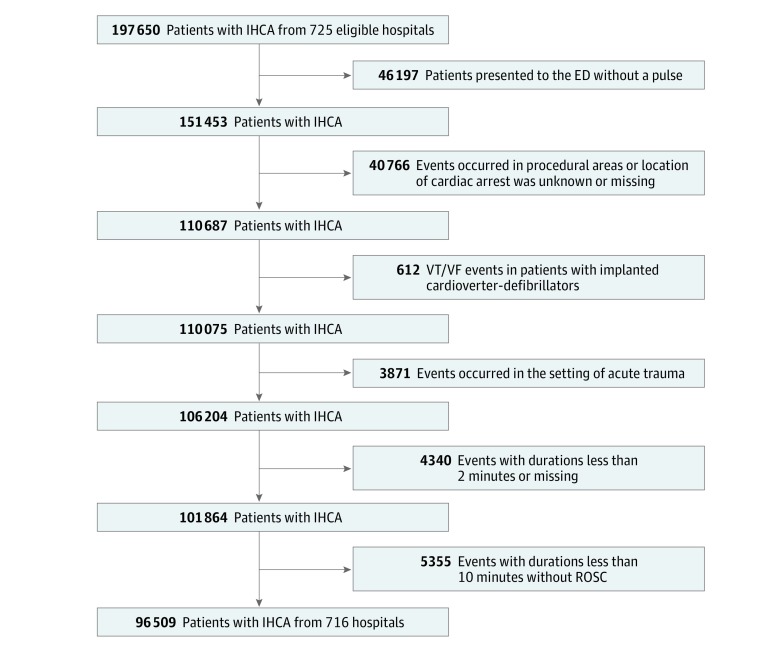
Population of Patients Used to Revalidate the UN10 Rule ED indicates emergency department; IHCA, in-hospital cardiac arrest; ROSC, return of spontaneous circulation; VF, ventricular fibrillation; and VT, ventricular tachycardia.

### Statistical Analysis

We assessed the UN10 rule’s performance using a 2 × 2 contingency table to compare observed and predicted survival to discharge; sensitivity, specificity, and predictive values were calculated using 95% CIs. Receiver operator curves are not presented in the original reports of the UN10 rule owing to the binary outcome of the decision rule^8^ and thus were excluded in our analysis. Statistical analyses were performed using Stata version 12.1 (StataCorp) and SAS version 9.4 (SAS Institute).

## Results

Baseline and intra-arrest characteristics of our cohort and those of the UN10 derivation (van Walraven et al^8^) and validation (van Walraven et al^7^) studies are presented in Table 1. In our study sample, 55 761 patients (57.8%) were men, and the mean (SD) age was 67.1 (15.3) years. Overall, a total of 52 293 patients (54.2%) achieved ROSC, 18 713 patients (19.4%) survived to discharge, and 16 134 patients (16.7%) were discharged with a favorable neurologic status.

**Table 1.** Intra-arrest Characteristics

Characteristic	No. (%)		
	UN10^a^ Derivation Study^8^	UN10^a^ Validation Study^7^	Current Study
Patients, No.	1077	1884	96 509
Arrests, No.	1077	2181	96 509
Age, y	67.9 (17-101)^b^	65.0 (64.3-65.7)^c^	67.1 (15.3)^d^
Men	616 (57.2)	993 (52.7)	55 761 (57.8)
Initial rhythm			
Pulseless VT or VF	338 (31.4)	481 (22.1)	20 120 (20.8)
Asystole or pulseless electrical activity	739 (68.6)	1700 (77.9)	76 389 (79.2)
Arrest witnessed	864 (80.2)	1721 (78.9)	74 780 (77.5)
ROSC	351 (32.6)^e^	1064 (48.8)^e^	52 293 (54.2)^f^
Mean duration of arrest, mean (SD), min	21.5 (17.4)	34 (0-225)^b^	22.5 (19.4)
Discharged			
Alive	103 (9.6)	327 (15.0)	18 713 (19.4)
With CPC score of 1 or 2	NA	NA	16 134 (16.7)

Abbreviations: CPC, cerebral performance category; NA, not applicable; ROSC, return of spontaneous circulation; VF, ventricular fibrillation; VT, ventricular tachycardia.

^a^ The UN10 rule is based on 3 variables: (1) unwitnessed arrest (not in person or by monitor), (2) a nonshockable initial rhythm (ie, pulseless electrical activity or asystole), and (3) no return of spontaneous circulation within 10 minutes of starting chest compressions.

^b^ Mean (range).

^c^ Mean (95% CI).

^d^ Mean (SD).

^e^ Defined as achieving ROSC for more than 1 hour.

^f^ Defined as achieving ROSC for more than 20 minutes.

In total, 15 838 patients with IHCA (16.4%) met all 3 UN10 criteria (Table 2). Of those, 1005 (6.3%) survived to discharge, and 754 (4.8%) survived with favorable neurologic status. By comparison, of the 80 671 patients (83.6%) who did not meet the UN10 rule, 17 708 (22.0%) survived to discharge, and 15 380 (19.1%) survived with favorable neurologic status. The percentage of patients meeting the UN10 rule (ie, predicting futile resuscitation) who actually survived in our study cohort was substantially higher than the initial derivation cohort (0%) and single-center validation cohort (1.1%). Notably, the positive predictive value was only 93.7% (95% CI, 93.3%-94.0%), which was lower than the initial derivation cohort (100% [95% CI, 97.5%-100%]) and validation cohort (98.9% [95% CI, 96.5%-99.7%]) (Table 3).

**Table 2.** Outcomes After In-Hospital Cardiac Arrest Stratified by UN10 Clinical Decision Rule^a^

UN10 Rule^a^ Predicts Futile Resuscitation	Patients Discharged Alive, No. (%)											
	Original UN10 Studies						Current Study					
	Derivation Study^8^			Validation Study^7^			Discharged Alive			CPC Score 1 or 2		
	Yes	No	Total, No.	Yes	No	Total, No.	Yes	No	Total, No.	Yes	No	Total, No.
No	103 (10.7)	855 (89.2)	958	324 (16.9)	1588 (83.1)	1912	17 708 (22.0)	62 963 (78.0)	80 671	15 380 (19.1)	65 291 (81.0)	80 671
Yes	0	119 (100)	119	3 (1.1)	266 (98.9)	269	1005 (6.3)	14 833 (93.7)	15 838	754 (4.8)	15 084 (95.2)	15 838
Total	103 (9.6)	974 (90.4)	1077	327 (15.0)	1854 (85.0)	2181	18 713 (19.4)	77 796 (80.6)	96 509	16 134 (16.7)	80 375 (83.3)	96 509

Abbreviation: CPC, cerebral performance category.

^a^ The UN10 rule is based on 3 variables: (1) unwitnessed arrest (not in person or by monitor), (2) a nonshockable initial rhythm (ie, pulseless electrical activity or asystole), and (3) no return of spontaneous circulation within 10 minutes of starting chest compressions.

**Table 3.** Sensitivity Analyses Using UN10 Clinical Decision Rule^a^

Analysis	% (95% CI)			
	Original UN10 Studies, Discharged Alive		Current Study	
	Derivation Study^8^	Validation Study^7^	Discharged Alive	CPC Score 1 or 2
Sensitivity	12.2 (10.3-14.4)	14.4 (12.4-16.0)	19.1 (18.8-19.3)	18.8 (18.5-19.0)
Specificity	100 (97.1-100)	99.1 (97.1-99.8)	94.6 (94.3-94.9)	95.3 (95.0-95.6)
Positive predictive value	100 (97.5-100)	98.9 (96.5-99.7)	93.7 (93.3-94.0)	95.2 (94.9-95.6)
Negative predictive value	10.8 (8.9-12.8)	17.0 (15.3-18.7)	22.0 (21.7-22.2)	19.1 (18.8-19.3)
Negative likelihood ratio	0.88	0.86	0.86	0.85

Abbreviation: CPC, cerebral performance category.

^a^ The UN10 rule is based on 3 variables: (1) unwitnessed arrest (not in person or by monitor), (2) a nonshockable initial rhythm (ie, pulseless electrical activity or asystole), and (3) no return of spontaneous circulation within 10 minutes of starting chest compressions.

## Discussion

The UN10 rule is a parsimonious CDR that demonstrated nearly perfect predictive ability to determine whether an ongoing resuscitation could be considered futile in initial studies. However, in a large contemporary cohort, the UN10 rule did not discriminate sufficiently to justify futility and discontinuation of resuscitative efforts for patients with IHCA. Given that 4.8% of patients meeting the UN10 rule had favorable neurologic survival and 6.3% survived to discharge, many patients and families may not consider resuscitative efforts futile at these levels.

Models using only intra-arrest variables to predict survival and guide resuscitative efforts remain limited. To our knowledge, most of the previous CDRs relying heavily on intra-arrest variables have not been validated using large national registries.^2,13,14,15^ The UN10 rule appears to be unique among current CDRs in that it relies solely on intra-arrest variables, which are often readily available during code situations, and it has now been validated in a large, national cohort.

Only recently have registries collected relevant data regarding quality of life measures at the time of discharge for patients following IHCA. Many of the prior models did not incorporate neurologic status into their calculations; however, this has been shown to be very important to survivors and has been included in more recent CDRs.^1,4^ In the current study, incorporation of neurologically intact survival was imperative because, although survival to discharge of patients meeting the UN10 rule for futility was 6.3%, if neurologically intact survival on discharge was lower than reported in this study (4.8%), then the CDR could have been more confidently reported as a tool for practitioners to terminate resuscitative efforts.

### Limitations

This study has several limitations that warrant further discussion. First, we used the GWTG-R database to validate the UN10 rule, and outcomes may differ at nonparticipating facilities. Second, the GWTG-R database is not a comprehensive data set and therefore does not include data such as the quality of chest compressions or duration of interruptions during cardiopulmonary resuscitation, which could alter results. Third, this study only assessed IHCAs with follow-up until discharge; therefore, it cannot be applied to out-of-hospital cardiac arrests and has no measure of outcomes following discharge. Fourth, treatment algorithms have rapidly changed (eg, increasing use of end-tidal carbon dioxide), survival rates for IHCA have increased, and use of palliative care practices in the creation of do not attempt resuscitation orders for patients have evolved since the original van Walraven et al^7,8^ studies in 1999 and 2001, which could explain some of the differences between our study and the original cohorts.^16^ Also, because the GWTG-R registry is a large national sample, it is unknown if hospitals included were using the UN10 rule from the original van Walraven et al^7,8^ studies for decision making following IHCA. Therefore, it is possible that this study could have artificially inflated the positive predictive value because health care professionals could have been using the rule to terminate resuscitation if a patient’s indexed cardiac arrest met the CDR criteria.

## Conclusions

In summary, when applied to a large, diverse patient population, we found that approximately 1 in 5 patients with an IHCA met the UN10 CDR. Rates of survival to discharge and favorable neurologic survival were approximately 6% and 5%, respectively, which suggests that the UN10 rule does not appear to have sufficient discrimination to be used to terminate acute resuscitations. However, given that it does identify patients whose probability of survival and favorable neurologic outcomes is significantly decreased, it could be used as an adjunct to decision making and potentially refined in the future to create a more predictive tool to aid in termination of resuscitative efforts following IHCA.
